# Understanding Anxiety and Knowledge Gaps Surrounding Laser Hair Removal: A Clinic-Based Cross-Sectional Study

**DOI:** 10.3390/healthcare14070835

**Published:** 2026-03-25

**Authors:** Amr Molla, Hamza Domlo, Mohammed Alraddadi

**Affiliations:** 1 Department of Medicine, College of Medicine, Taibah University, Madinah 42353, Saudi Arabia; 2 University Medical Center, Taibah University, Madinah 42353, Saudi Arabia; hamza_domlo@hotmail.com; 3 Department of Medicine, College of Medicine, University of Tabuk, Tabuk 71491, Saudi Arabia; dr.m.o.alraddadi@gmail.com

**Keywords:** laser hair removal, anxiety, knowledge, misconceptions, Saudi Arabia

## Abstract

**Background:** Laser hair removal (LHR) is a widely used cosmetic procedure; however, misconceptions and anxiety regarding its safety and effectiveness remain common and may influence treatment decisions. **Objective:** The aim of this study was to assess attitudes, misconceptions, and anxiety related to LHR among adult dermatology clinic attendees in Saudi Arabia and to identify factors associated with LHR-related anxiety and knowledge levels. **Methods:** A cross-sectional clinic-based survey was conducted among adults in Saudi Arabia between August and December 2025 using a structured Arabic/English questionnaire. Data collected included sociodemographic characteristics, LHR-related anxiety measured on a visual analog scale (VAS), and LHR knowledge. Anxiety was analyzed as both a continuous score and a categorical variable, while knowledge was scored using an 11-item questionnaire. Associations were examined using chi-square tests, and independent predictors were assessed using multinomial logistic regression. **Results:** A total of 400 participants were included (mean age 24.5 ± 7.9 years; 89.5% female). Overall anxiety was low (mean VAS 2.5 ± 2.9), although 32.2% reported moderate-to-severe anxiety. Adequate knowledge was observed in 39.5% of participants. In adjusted analyses, adequate knowledge was independently associated with lower likelihood of severe anxiety. Female sex and Saudi nationality were strong predictors of higher knowledge levels. **Conclusions:** In this clinic-based sample, LHR-related anxiety was generally low, although misconceptions persisted in a substantial proportion of participants. Improving patient knowledge through targeted, evidence-based education may help reduce anxiety, support realistic expectations, and promote informed decision-making regarding LHR.

## 1. Introduction

Unwanted hair is a common aesthetic concern for both women and men, spanning a spectrum from minimal, barely visible hair to clinically bothersome or excessive growth. While increased facial or body hair can occasionally indicate an underlying medical condition or driver, such as endocrine imbalance or other systemic conditions, most presentations reflect normal physiologic variation rather than pathology [1,2]. When hair growth is prominent in cosmetically sensitive sites, it may adversely affect self-perception and confidence and has been linked to psychological burden, poorer quality of life, strained social interactions, social avoidance, and depressive symptoms [3,4].

Despite their widespread use, conventional hair-removal practices—including shaving, waxing, plucking, and chemical depilatories—offer only short-lived control of hair growth and often require frequent repetition. Although these methods are accessible and relatively low-cost, they may be associated with discomfort and local reactions such as irritation, swelling, blistering, crusting, and post-inflammatory pigmentary changes, and their convenience and effectiveness vary substantially between individuals [5,6]. By contrast, laser and other light-based technologies have become the leading options for durable hair reduction and are among the most frequently performed cosmetic procedures worldwide. Their clinical effect relies on selective photothermolysis, in which light energy preferentially absorbed by follicular melanin is converted to heat, producing targeted thermal injury to the follicle while minimizing damage to surrounding skin structures [7,8]. Multiple laser and non-laser light-based platforms are used for long-term hair reduction, including diode lasers (810 nm), Alexandrite lasers (755 nm), neodymium-doped yttrium aluminum garnet (Nd:YAG) lasers (1064 nm), and intense pulsed light (IPL, 590–1200 nm), which is a non-coherent light technology rather than a laser system [7,8].

Overall, LHR is regarded as a well-tolerated procedure with adverse events that are typically mild and transient [9]. Nevertheless, concerns about safety, pain, skin damage, and long-term effects may still provoke anxiety or avoidance, particularly when individuals rely on incomplete or inaccurate information. In the broader aesthetic medicine literature, pre-procedural anxiety and negative risk appraisal have been recognized as important influences on patients’ decision-making, expectations, and satisfaction, and psychological factors such as appearance-related concern, low confidence in procedural safety, and catastrophic interpretation of adverse outcomes may amplify perceived threat beyond objective risk. Empirical studies in cosmetic and aesthetic settings have similarly shown that anxiety-related symptoms, social appearance anxiety, and lower educational attainment may shape willingness to pursue procedures and perceptions of harm [10,11,12]. Against this background, misconceptions surrounding LHR are clinically important because they may influence whether individuals seek treatment, how they interpret normal transient reactions, and how they respond to counseling. Only a limited number of studies have specifically examined unfavorable perceptions and misconceptions regarding LHR [13,14]. Moreover, evidence from Saudi Arabia addressing public beliefs and perceptions about LHR remains limited [15], highlighting the need for additional research to characterize prevailing misconceptions and to support accurate counseling, health literacy, and informed decision-making.

Accordingly, the objective of this study was to assess attitudes, misconceptions, and anxiety related to LHR among adult dermatology clinic attendees in Saudi Arabia and to identify factors associated with LHR-related anxiety and knowledge levels.

## 2. Materials and Methods

### 2.1. Study Design and Sampling

This cross-sectional, questionnaire-based study was conducted over approximately five months, from August 2025 to December 2025, among adult dermatology clinic attendees in Saudi Arabia. Participants were recruited using a convenience sampling approach during routine dermatology clinic visits, where they completed a structured self-administered questionnaire in Arabic or English assessing knowledge, attitudes, and misconceptions regarding LHR. Although recruitment occurred in clinical settings, the questionnaire addressed general knowledge and perceptions about LHR and was not limited to individuals actively seeking laser hair removal.

The study population included Saudi nationals and non-Saudi residents of either sex who are currently living in Saudi Arabia, aged 18 years or older at the time of participation, able to read and understand Arabic or English, mentally competent, and willing to provide informed consent. Individuals younger than 18 years, those not currently residing in Saudi Arabia, persons with cognitive or mental conditions that impair their ability to understand or complete the questionnaire, individuals unable to comprehend either Arabic or English, and those who decline or are unable to provide informed consent were excluded.

A minimum target sample size of 400 participants was estimated using the Raosoft^®^ online sample size calculator (Raosoft, Inc., Seattle, WA, USA), assuming a 95% confidence level and a 5% margin of error.

### 2.2. Data Collection

The questionnaire consisted of two main components. The first component collected sociodemographic information, including age, sex, nationality, educational level, marital status, residence, occupation, and self-perceived monthly income categorized as low, low–moderate, high–moderate, or high.

The second component assessed participants’ anxiety and knowledge related to laser hair removal (LHR). LHR-related anxiety was measured using a Visual Analog Scale (VAS), with scores ranging from 0 (no anxiety) to 10 (extreme anxiety) [16]. Knowledge and misconceptions regarding LHR were evaluated using a self-administered questionnaire comprising 11 statements derived from commonly encountered questions in routine clinical practice. Responses to each item were recorded on a 3-point scale (Yes/No/Do not know). Total scores ranged from 0 to 22, with higher scores indicating more adequate knowledge and fewer misconceptions. The internal consistency of the knowledge questionnaire was high (Cronbach’s alpha = 0.858).

The knowledge questionnaire consisted of 11 statements addressing common beliefs and misconceptions about LHR, including expected permanence of hair reduction, perceived radiation exposure, safety during pregnancy and lactation, use in children, cancer-related concerns, infertility concerns, lymph node damage, the need for protective eyewear, and post-procedure sun protection. Responses to each item were recorded on a 3-point scale (Yes/No/Do not know), and the full item list is presented in Supplementary Table S1. Items were scored according to correctness, with more accurate responses assigned higher values, yielding a total score range of 0–22. Higher scores indicated better knowledge and fewer misconceptions. For descriptive analysis, total scores were categorized into inadequate, moderate, and adequate knowledge levels using predefined score bands based on the observed score distribution and interpretability of the scale.

### 2.3. Statistical Analysis

Statistical analyses were performed using IBM SPSS Statistics for Windows, Version 26.0 (IBM Corp., Armonk, NY, USA). Categorical variables are presented as frequencies and percentages. Continuous variables were assessed for normality and summarized as mean (SD) when approximately normally distributed, or median (IQR) with minimum–maximum when non-normally distributed. Anxiety related to laser hair removal (LHR) and LHR knowledge were analyzed both as continuous scores and as categorical outcomes (anxiety: mild/moderate/severe; knowledge: inadequate/moderate/adequate). The continuous VAS anxiety score was treated as the primary representation of anxiety, while categorical groupings (mild, moderate, and severe) were used as secondary descriptive and regression outcomes to facilitate clinical interpretation of anxiety severity. For descriptive categorization, VAS scores were grouped into mild, moderate, and severe anxiety using pragmatic ordinal cutoffs across the 0–10 scale. Bivariate associations between participant characteristics and categorical anxiety/knowledge levels were examined using the chi-square test (or Fisher’s exact test when expected cell counts were <5). Multivariable multinomial logistic regression models were used to identify independent predictors of (1) anxiety level (reference category: severe) and (2) knowledge level (reference category: inadequate), adjusting for sociodemographic covariates (gender, age, nationality, education, marital status, residence, occupation, and income level). Adjusted odds ratios (aOR) with 95% confidence intervals (CI) are reported. Two-tailed *p*-values < 0.05 were considered statistically significant. To assess model robustness, sparse categories were retained because they represented clinically relevant participant subgroups, but their corresponding estimates were interpreted cautiously, particularly for the smaller male and non-Saudi groups. Multicollinearity among predictor variables was assessed prior to model fitting and was not considered to materially affect model interpretation. Reference categories were selected a priori on the basis of clinical interpretability and because they represented either the largest or most conceptually appropriate comparison group.

### 2.4. Ethical Considerations

The study protocol was reviewed and approved by the Institutional Review Board (IRB) committee of the General Directorate of Health Affairs in Madinah (reference number 25-058; approval date: 7 August 2025). Participation was entirely voluntary. Before completing the questionnaire, all potential participants were provided with an information sheet explaining the study objectives, procedures, potential risks and benefits, and their rights, including the right to withdraw at any time without penalty. Informed consent was obtained prior to enrollment. Data were collected anonymously, stored securely, and used solely for research purposes. Confidentiality was maintained at all stages of the research process and after completion of the study, in accordance with institutional guidelines and the principles of the Declaration of Helsinki.

## 3. Results

### 3.1. Sociodemographic Characteristics

Table 1 summarizes the sociodemographic profile of the 400 participants. Most were female (358/400, 89.5%), with a mean age of 24.45 ± 7.94 years (median 22 [IQR 5], range 18–55). The majority were Saudi nationals (366/400, 91.5%) and lived in urban areas (363/400, 90.8%). Regarding education, 52.5% were graduates and 1.0% postgraduates, while 46.5% were undergraduates. Most participants were single (318/400, 79.5%) and students (282/400, 70.5%). Economically, 85.0% were in the low and low–moderate income categories (low: 41.8%; low–moderate: 43.2%) (Table 1).

### 3.2. Anxiety and Knowledge Levels Related to Laser Hair Removal

Overall anxiety was low, with a mean VAS anxiety score of 2.51 ± 2.87 (median 2 [IQR 5], range 0–10). Most participants reported mild anxiety (271/400, 67.8%), whereas 76/400 (19.0%) had moderate anxiety and 53/400 (13.2%) had severe anxiety. Knowledge scores were generally in the moderate-to-high range, with a mean score of 14.8 ± 4.01 (median 16 [IQR 6], range 0–22). Overall, 158/400 participants (39.5%) demonstrated adequate knowledge, 171/400 (42.8%) had moderate knowledge, and 71/400 (17.8%) had inadequate knowledge (Table 2).

### 3.3. Factors Associated with Anxiety Level Related to Laser Hair Removal

Table 3 shows that LHR-related anxiety level was significantly associated with several variables. Gender was strongly associated with anxiety (*p* = 0.007), with males reporting a higher proportion of severe anxiety than females (26.2% vs. 11.7%). Nationality was also significant (*p* = 0.03), as non-Saudi participants had a higher proportion of severe anxiety than Saudi participants (26.5% vs. 12.0%). Marital status was associated with anxiety (*p* = 0.039), with married participants reporting severe anxiety more frequently than unmarried participants (22.1% vs. 11.1%). Income level showed a significant association (*p* = 0.022); the low-income group had the highest proportion of severe anxiety (18.0%), while the low–moderate group had the lowest (9.8%). Finally, knowledge level was strongly associated with anxiety (*p* = 0.001): participants with adequate knowledge had the lowest prevalence of severe anxiety (5.7%) compared with those with moderate (20.5%) or inadequate knowledge (12.7%) (Table 3).

### 3.4. Factors Associated with Knowledge Level Related to Laser Hair Removal

As shown in Table 4, LHR knowledge level varied significantly by several sociodemographic factors. Gender demonstrated the strongest association (*p* < 0.001), with females more likely to have adequate knowledge than males (43.3% vs. 7.1%), whereas nearly half of males were classified as having inadequate knowledge (47.6%). Nationality was also strongly associated with knowledge level (*p* < 0.001); Saudi participants had substantially higher adequate knowledge (42.9%) than non-Saudi participants (2.9%), among whom most had moderate knowledge (70.6%). Education level was significantly associated with knowledge (*p* = 0.049), with higher adequate knowledge in the graduate/postgraduate group (43.0%) compared with the undergraduate group (35.5%). Residence was also significant (*p* = 0.044), with urban residents showing the highest adequate knowledge (41.0%) compared with rural (20.0%) and suburban participants (29.4%). Income level was strongly associated with knowledge (*p* < 0.001), with adequate knowledge highest in the low-income category (46.7%) and lowest in the high-income category (27.8%) (Table 4).

### 3.5. Multinomial Logistic Regression of Factors Associated with LHR-Related Anxiety Level

After adjustment for sociodemographic variables and knowledge level, adequate LHR knowledge was independently associated with lower likelihood of severe anxiety. Compared with participants with moderate knowledge (reference), those with adequate knowledge had significantly higher odds of being in the mild anxiety category rather than severe (aOR = 4.17, *p* = 0.001) and higher odds of being in the moderate category rather than severe (aOR = 3.38, *p* = 0.016).

Female sex was also associated with higher odds of mild vs. severe anxiety (aOR = 3.43, *p* = 0.013). In contrast, married participants had significantly lower odds of being classified as mild vs. severe (aOR = 0.17, *p* = 0.006) and moderate vs. severe (aOR = 0.11, *p* = 0.003), indicating a higher likelihood of being in the severe anxiety group relative to unmarried participants. Additionally, participants in the low-income group had lower odds of mild vs. severe anxiety compared with the low–moderate-income reference group (aOR = 0.33, *p* = 0.004), suggesting greater relative representation in the severe anxiety category (Table 5).

### 3.6. Multinomial Logistic Regression of Factors Associated with LHR Knowledge Level

In the adjusted multinomial model, female gender was the strongest independent predictor of higher knowledge. Compared with males, females had markedly higher odds of having adequate vs. inadequate knowledge (aOR = 17.15, *p* < 0.001) and higher odds of moderate vs. inadequate knowledge (aOR = 2.75, *p* = 0.018).

Non-Saudi nationality was independently associated with substantially lower odds of adequate vs. inadequate knowledge (aOR = 0.012, *p* < 0.001). Income level also remained significant: relative to the low–moderate-income reference group, participants in the low-income group had higher odds of adequate vs. inadequate knowledge (aOR = 7.18, *p* < 0.001) and moderate vs. inadequate knowledge (aOR = 4.06, *p* = 0.001).

Finally, compared with severe anxiety (reference), mild anxiety showed a borderline association with higher odds of adequate vs. inadequate knowledge (aOR = 3.14, *p* = 0.050), whereas moderate anxiety was not significantly associated with knowledge category (Table 6).

## 4. Discussion

### 4.1. Principal Findings

In this cross-sectional survey of adults in Saudi Arabia (*n* = 400), we assessed attitudes and misconceptions toward laser hair removal (LHR) and factors associated with LHR-related anxiety and knowledge. Overall anxiety was low (VAS mean ≈ 2.5/10), although a subgroup reported moderate-to-severe anxiety. Knowledge was generally moderate-to-high, yet a notable proportion had inadequate knowledge. In bivariate analyses, anxiety differed by gender, nationality, marital status, income, and knowledge level, whereas knowledge differed by gender, nationality, education, residence, and income. In adjusted multinomial models, adequate knowledge was independently associated with lower likelihood of severe anxiety, and female sex was associated with higher odds of mild versus severe anxiety. Married participants and those in the low-income group were relatively more represented in the severe anxiety category. Knowledge level was independently associated with female sex and Saudi nationality, while low income was associated with higher odds of both adequate and moderate knowledge; mild anxiety showed a borderline association with adequate knowledge.

Collectively, these findings suggest that misconceptions and anxiety around LHR coexist even in a predominantly young, educated sample, and that knowledge is meaningfully linked to anxiety severity. However, the interpretation should remain cautious. First, the cross-sectional design precludes causal inference: higher knowledge could reduce anxiety, but the reverse is also plausible (e.g., those who are less anxious may seek information more readily), and unmeasured factors such as prior LHR exposure, personal or peer adverse experiences, and information sources (clinicians vs. social media) may influence both outcomes. Second, multiple bivariate comparisons increase the chance of type I error; thus, greater weight should be placed on associations that are consistent across bivariate and multivariable models and that are clinically plausible. Third, some statistically significant findings—particularly those related to income—may reflect residual confounding or misclassification (self-perceived income categories may correlate imperfectly with socioeconomic position in a young, student-heavy sample).

### 4.2. Interpretation in Relation to Previous Literature

When compared with prior literature, our results are broadly concordant with evidence that misconceptions about lasers remain common and that knowledge varies systematically by sociodemographic characteristics. In a large survey of dermatology clinic patients in Riyadh, AlGhamdi and Moussa documented substantial misconceptions about lasers (e.g., uncertainty about the nature of lasers, beliefs that lasers are unsafe in pregnancy or harmful to children, and concerns about cancer), with negative attitudes more frequent among men and those with lower education—supporting the notion that targeted education can address exaggerated fears and misinformation [15]. Similarly, Vachiramon and McMichael’s survey in African American participants found notable knowledge gaps and concern regarding LHR in darker skin types (only about half knew LHR could be performed in dark skin), and they observed that female sex and higher educational background were associated with greater willingness to consider LHR—paralleling our finding that female sex is strongly associated with higher knowledge categories [13]. More recently, the Omani study by Al Hatmi and colleagues (dermatology clinic attendees) also reported a general lack of knowledge and misconceptions, with higher knowledge among those who had used LHR and higher knowledge in women and those with college education or above, while not observing differences by employment status or monthly income—findings that are directionally similar regarding sex and education but differ from our income association [14]. In a larger and more recent clinic-based survey from Turkey (*n* = 1002), Agaoglu et al. similarly found that participants who had previously undergone LHR had higher knowledge scores and higher health literacy. They also reported that greater knowledge, adequate health literacy, and lower levels of concern were independently associated with favoring LHR, supporting the clinical plausibility of a link between knowledge and anxiety-related perceptions [17]. These differences likely reflect variation in sampling frames (general population surveys vs. clinic-based samples), demographic composition (our sample was younger and predominantly female), and the role of direct LHR experience (a strong driver of knowledge in the Omani study but not explicitly modeled in our analyses).

From a mechanistic perspective, the inverse association between adequate knowledge and severe anxiety is clinically plausible: clearer understanding of LHR safety, expected sensations, realistic outcomes, and adverse-event profiles may reduce uncertainty and catastrophic interpretations. In contrast, persistent misconceptions—particularly fears of long-term harm—may heighten anxiety despite the procedure’s generally favorable safety profile. Differences by gender and nationality may reflect variation in access to dermatologic counseling, exposure to cosmetic procedures, culturally shaped information networks, and reliance on non-medical sources. The higher relative representation of married participants in the severe-anxiety reference category should be interpreted cautiously, as it may reflect differences in risk perception or prior experiences and residual confounding (e.g., age or life context) rather than a direct effect of marital status. Similarly, income-related patterns require caution because “low income” in a student-dominant sample may function as a proxy for younger age or greater digital engagement rather than reduced health-information access.

### 4.3. Clinical Implications and Future Research Directions

From a clinical perspective, these findings suggest that misconceptions about LHR are not simply informational gaps, but may directly influence risk perception, treatment hesitancy, and interpretation of expected procedural effects. In routine dermatology practice, this underscores the importance of counseling that clearly explains realistic treatment outcomes, particularly that LHR usually provides long-term hair reduction rather than guaranteed permanent eradication. Counseling should also distinguish expected transient effects, such as mild erythema, perifollicular edema, or temporary discomfort, from warning signs that warrant reassessment, including blistering, persistent pigmentary change, or unexpected burns. In addition, clinicians should individualize discussion of risk according to skin phototype, device type, wavelength selection, and operator expertise, since these factors influence both effectiveness and adverse-event profiles. Such counseling may help reduce anxiety by replacing vague or exaggerated concerns with accurate and actionable information.

The observed association between lower knowledge and more severe anxiety also raises the possibility that, in some individuals, perceived threat related to laser exposure may be influenced by cognitive biases rather than objective risk appraisal alone. In the broader anxiety literature, overestimation of threatening outcomes has been recognized as an important cognitive mechanism. Experimental work has shown that cognitive techniques can reduce exaggerated probability estimates of negative events in both high- and low-anxiety individuals. For example, Gangemi and colleagues demonstrated that structured cognitive approaches such as the Pie Technique, Cumulative Probability, and the Inverted Pyramid reduced the perceived likelihood of threatening events, with effects maintained at short-term follow-up [18]. Although this work was not conducted in an LHR context, it supports the conceptual relevance of probability overestimation, fear amplification, and catastrophizing when considering anxiety related to generally safe medical or cosmetic procedures.

Future research should therefore move beyond descriptive assessment of misconceptions and incorporate psychological constructs such as health literacy, intolerance of uncertainty, catastrophizing, social appearance anxiety, and perceived procedural threat. Longitudinal studies would help determine whether greater knowledge reduces anxiety over time or whether less anxious individuals are simply more likely to seek accurate information. Interventional studies are also warranted to assess whether brief educational or cognitively informed counseling strategies can reduce exaggerated fears, improve risk understanding, and support more informed decision-making regarding LHR. In addition, qualitative studies may help identify culturally specific beliefs, misinformation pathways, and communication needs that shape public attitudes toward light-based cosmetic procedures.

### 4.4. Strengths and Limitations

This study has several strengths. It addresses an underexplored topic in Saudi Arabia, uses a structured questionnaire with high internal consistency, and applies multivariable modeling to examine factors associated with both anxiety and knowledge. These features strengthen the internal coherence of the findings and provide clinically relevant insight into how misconceptions and anxiety may interact in the context of a commonly performed cosmetic procedure.

At the same time, several limitations should be acknowledged. First, the use of clinic-based convenience sampling limits generalizability and may have resulted in overrepresentation of younger, female, urban, and more educated participants, potentially inflating knowledge estimates and underestimating anxiety prevalence. This sample composition is also important when interpreting the apparently higher knowledge observed among lower-income participants, as in this setting low income may reflect student status or younger age rather than socioeconomic disadvantage in the usual sense. Therefore, extrapolation to men, older adults, rural populations, and non-Saudi groups should be made cautiously. Second, the study relied on self-reported measures, which may be subject to recall bias or social desirability bias. Third, residual confounding remains possible, particularly because potentially relevant variables such as prior LHR use, prior adverse experiences, skin phototype, health literacy, and information sources were not directly measured. Finally, the multiplicity of analyses increases the possibility of spurious associations, and some findings—particularly those related to income—should be interpreted as hypothesis-generating rather than definitive.

## 5. Conclusions

In this clinic-based cross-sectional sample of adults in Saudi Arabia, LHR-related anxiety was generally low, although a meaningful subgroup reported moderate-to-severe anxiety and a notable proportion demonstrated inadequate knowledge. Knowledge level showed a consistent relationship with anxiety severity, with adequate knowledge independently associated with a lower likelihood of severe anxiety, underscoring the importance of accurate, accessible information. Clear disparities by gender and nationality suggest that misconceptions and informational needs are not uniform across participants. These findings support a role for dermatology-led, culturally appropriate education—delivered in clinics and through trusted digital platforms—to improve expectations, correct misconceptions, and support informed decision-making regarding LHR. Future studies using more representative sampling and longitudinal or interventional designs are warranted to clarify causality and evaluate targeted educational strategies that improve patient knowledge and experience with LHR.

## Figures and Tables

**Table 1:** Sociodemographic characteristics of the study participants (N = 400).

Variable	Category	N	%
Gender	Female	358	89.5
	Male	42	10.5
Age (Years)	Mean (SD)	24.45 (7.94)	
	Median (IQR)	22 (5)	
	Min–Max	18–55	
Nationality	Saudi	366	91.5
	Egyptian	7	1.8
	Nigerian	1	0.2
	Sudan	2	0.5
	Syrian	6	1.5
	Yemeni	18	4.5
Education	Graduate	210	52.5
	Postgraduate	4	1
	Undergraduate	186	46.5
Marital status	Single	318	79.5
	Married	77	19.2
	Divorced	3	0.8
	Separated	1	0.2
	Widow	1	0.2
Residence	Urban	363	90.8
	Rural	20	5
	Suburban	17	4.2
Occupation	Student	282	70.5
	Employed	49	12.2
	Unemployed	69	17.2
Income level	Low	167	41.8
	Low–moderate	173	43.2
	High–moderate	24	6
	High	36	9

Values are presented as N (%). Percentages were calculated using the total sample size (N = 400).

**Table 2:** Anxiety and knowledge regarding laser hair removal among study participants.

Visual Analog Scale (VAS) anxiety score	Mean (SD)	2.51 (2.9)
	Median (IQR)	2 (5)
	Min–Max	0–10
Level of anxiety related to LHR [N (%)]	Mild	271 (67.8)
	Moderate	76 (19)
	Severe	53 (13.2)
Knowledge score	Mean (SD)	14.8 (4)
	Median (IQR)	16 (6)
	Min–Max	0–22
Level of knowledge related to LHR [N (%)]	Inadequate	71 (17.8)
	Moderate	171 (42.8)
	Adequate	158 (39.5)

Values are presented as N (%). Percentages were calculated using the total sample size (N = 400).

**Table 3:** Associations between participant characteristics, knowledge level, and anxiety related to laser hair removal.

Variable	Category	Anxiety Level Related to LHR N (%) ^a^			*p*-Value ^b^
		Mild	Moderate	Severe	
Gender	Female	251 (70.1)	65 (18.2)	42 (11.7)	0.007
	Male	20 (47.6)	11 (26.2)	11 (26.2)	
Age (years)	18–25	218 (70.1)	56 (18)	37 (11.9)	0.15
	>25	53 (59.6)	20 (22.5)	16 (18)	
Nationality	Saudi	254 (69.4)	68 (18.6)	44 (12)	0.03
	Non-Saudi	17 (50)	8 (23.5)	9 (26.5)	
Education	Graduated/postgraduate	151 (70.6)	39 (18.2)	24 (11.2)	0.346
	Undergraduate	120 (64.5)	37 (19.9)	29 (15.6)	
Marital status	Married	47 (61)	13 (16.9)	17 (22.1)	0.039
	Unmarried	224 (69.3)	63 (19.5)	36 (11.1)	
Residence	Rural	15 (75)	3 (15)	2 (10)	0.824
	Suburban	13 (76.5)	3 (17.6)	1 (5.9)	
	Urban	243 (66.9)	70 (19.3)	50 (13.8)	
Occupation	Employed	33 (67.3)	10 (20.4)	6 (12.2)	0.934
	Unemployed	44 (63.8)	14 (20.3)	11 (15.9)	
	Student	194 (68.8)	52 (18.4)	36 (12.8)	
Income level	Low	102 (61.1)	35 (21)	30 (18)	0.022
	Low–moderate	128 (74)	28 (16.2)	17 (9.8)	
	High–moderate	13 (54.2)	9 (37.5)	2 (8.3)	
	High	28 (77.8)	4 (11.1)	4 (11.1)	
LHR Knowledge level	Adequate	121 (76.6)	28 (17.7)	9 (5.7)	0.001
	Moderate	105 (61.4)	31 (18.1)	35 (20.5)	
	Inadequate	45 (63.4)	17 (23.9)	9 (12.7)	

^a^ Values are presented as N (%); percentages were calculated by row. ^b^ *p*-values were calculated using chi-square or Fisher’s exact test, as appropriate; *p* < 0.05 was considered statistically significant.

**Table 4:** Associations between participant characteristics and knowledge level regarding laser hair removal.

Variables	Category	LHR Knowledge Level N (%) ^a^			*p*-Value ^b^
		Adequate	Moderate	Inadequate	
Gender	Female	155 (43.3)	152 (42.5)	51 (14.2)	<0.001
	Male	3 (7.1)	19 (45.2)	20 (47.6)	
Age (years)	18–25	126 (40.5)	124 (39.9)	61 (19.6)	0.055
	>25	32 (36)	47 (52.8)	10 (11.2)	
Nationality	Saudi	157 (42.9)	147 (40.2)	62 (16.9)	<0.001
	Non-Saudi	1 (2.9)	24 (70.6)	9 (26.5)	
Education	Graduated/postgraduate	92 (43)	93 (43.5)	29 (13.6)	0.049
	Undergraduate	66 (35.5)	78 (41.9)	42 (22.6)	
Marital status	Married	33 (42.9)	32 (41.6)	12 (15.6)	0.757
	Unmarried	125 (38.7)	139 (43)	59 (18.3)	
Residence	Rural	4 (20)	9 (45)	7 (35)	0.044
	Suburban	5 (29.4)	6 (35.3)	6 (35.3)	
	Urban	149 (41)	156 (43)	58 (16)	
Occupation	Employed	25 (51)	16 (32.7)	8 (16.3)	0.111
	Unemployed	26 (37.7)	36 (52.2)	7 (10.1)	
	Student	107 (37.9)	119 (42.2)	56 (19.9)	
Income level	Low	78 (46.7)	78 (46.7)	11 (6.6)	<0.001
	Low–moderate	58 (33.5)	70 (40.5)	45 (26)	
	High–moderate	12 (50)	10 (41.7)	2 (8.3)	
	High	10 (27.8)	13 (36.1)	13 (36.1)	

^a^ Values are presented as N (%); percentages were calculated by row. ^b^ *p*-values were calculated using chi-square or Fisher’s exact test, as appropriate; *p* < 0.05 was considered statistically significant.

**Table 5:** Multinomial logistic regression analysis of factors associated with anxiety related to laser hair removal.

Variable	aOR	95% CI	*p*-Value
Mild [ref: severe]			
Gender: Female [ref: Male]	3.183	1.221–8.296	0.018
Nationality: Non-Saudi [ref: Saudi]	0.568	0.174–1.852	0.348
Age: 18–25 years [ref: >25]	1.315	0.490–3.526	0.586
Education: Graduated/post-graduated [ref: Undergraduate]	2.026	0.951–4.319	0.067
Marital status: Married [ref: unmarried]	0.155	0.045–0.541	0.003
Residence [ref: Urban]			
Rural	1.124	0.224–5.647	0.888
Suburban	5.311	0.539–52.302	0.152
Occupation [ref: Unemployed]			
Employed	0.449	0.106–1.913	0.279
Student	0.337	0.088–1.298	0.114
Income level [ref: Low–moderate]			
High	0.847	0.235–3.055	0.800
High–moderate	0.831	0.132–5.236	0.843
Low	0.327	0.156–0.684	0.003
LHR knowledge level [ref: Moderate]			
Adequate	4.155	1.807–9.550	<0.001
Inadequate	1.635	0.647–4.132	0.298
Moderate [ref: severe]			
Gender: Female [ref: Male]	1.475	0.495–4.395	0.485
Nationality: Non-Saudi [ref: Saudi]	0.962	0.244–3.789	0.956
Age: 18–25 years [ref: >25]	0.802	0.264–2.438	0.697
Education: Graduate/post-graduate [ref: Undergraduate]	1.461	0.609–3.506	0.396
Marital status: Married [ref: unmarried]	0.102	0.024–0.430	0.002
Residence [ref: Urban]			
Rural	0.839	0.119–5.927	0.860
Suburban	3.640	0.312–42.409	0.302
Occupation [ref: Unemployed]			
Employed	0.438	0.081–2.378	0.339
Student	0.300	0.068–1.325	0.112
Income level [ref: Low–moderate]			
High	0.674	0.133–3.406	0.633
High–moderate	4.662	0.629–34.575	0.132
Low	0.613	0.260–1.443	0.262
LHR knowledge level [ref: Moderate]			
Adequate	3.595	1.385–9.333	0.009
Inadequate	2.556	0.892–7.320	0.080

Abbreviations: aOR, adjusted odds ratio; CI, confidence interval; ref, reference. Note: *p* values were derived from multinomial logistic regression models adjusted for the covariates shown.

**Table 6:** Multinomial logistic regression analysis of factors associated with knowledge regarding laser hair removal.

Variable	aOR	95% CI	*p*-Value
Adequate [ref: Inadequate]			
Gender: Female [ref: Male]	18.444	4.811–70.706	<0.001
Nationality: Non-Saudi [ref: Saudi]	0.012	0.001–0.118	<0.001
Age: 18–25 years [ref: >25]	0.534	0.178–1.605	0.264
Education: Graduated/post-graduated [ref: Undergraduate]	1.446	0.675–3.096	0.342
Marital status: Married [ref: unmarried]	0.922	0.279–3.052	0.895
Residence [ref: Urban]			
Rural	0.331	0.082–1.331	0.119
Suburban	0.444	0.112–1.767	0.249
Occupation [ref: Unemployed]			
Employed	1.210	0.279–5.259	0.799
Student	0.600	0.161–2.237	0.447
Income level [ref: Low–moderate]			
High	0.394	0.142–1.095	0.074
High–moderate	1.733	0.299–10.052	0.540
Low	7.416	3.124–17.602	<0.001
LHR Anxiety level [ref: Severe]			
Mild	2.938	0.964–8.954	0.058
Moderate	1.491	0.426–5.225	0.532
Moderate [ref: Inadequate]			
Gender: Female [ref: Male]	2.948	1.283–6.775	0.011
Nationality: Non-Saudi [ref: Saudi]	0.375	0.123–1.143	0.085
Age: 18–25 years [ref: >25]	0.437	0.151–1.263	0.126
Education: Graduated/post-graduated [ref: Undergraduate]	1.638	0.799–3.356	0.178
Marital status: Married [ref: unmarried]	0.401	0.123–1.300	0.128
Residence [ref: Urban]			
Rural	0.539	0.170–1.714	0.295
Suburban	0.373	0.105–1.323	0.127
Occupation [ref: Unemployed]			
Employed	0.533	0.125–2.277	0.396
Student	0.480	0.136–1.693	0.254
Income level [ref: Low–moderate]			
High	0.552	0.218–1.402	0.212
High–moderate	2.603	0.449–15.104	0.286
Low	4.336	1.937–9.705	<0.001
LHR Anxiety level [ref: Severe]			
Mild	0.740	0.297–1.845	0.519
Moderate	0.426	0.151–1.198	0.106

Abbreviations: aOR, adjusted odds ratio; CI, confidence interval; ref, reference. Note: *p* values were derived from multinomial logistic regression models adjusted for the covariates shown.

## Data Availability

The data that support the findings of this research are available from the corresponding author upon reasonable request. Due to legal and ethical considerations, the data cannot be made publicly available. Requests for data access should be directed to the Corresponding Author.

## Supplementary Materials

The following supporting information can be downloaded at: https://www.mdpi.com/article/10.3390/healthcare14070835/s1, Table S1: Distribution of participants’ responses to the 11-item LHR knowledge questionnaire.
