# Supplementary material for: Understanding Anxiety and Knowledge Gaps Surrounding Laser Hair Removal: A Clinic-Based Cross-Sectional Study
Source: Healthcare (Basel). 2026 Mar 25;14(7):835. doi: 10.3390/healthcare14070835 (PMC13073951; doi:10.3390/healthcare14070835)
Supplement: Supplementary file 1 [file healthcare-14-00835-s001.zip › healthcare-4164354-supplementary.pdf]

Supplementary Table S1. Distribution of participants' responses to the 11-item LHR knowledge questionnaire

Item	Yes n (%)	No n (%)	Do not know n (%)
1. Laser epilation could be used to removal of unwanted hair permanently (T)	330 (82.5)	20 (5.0)	50 (12.5)
2. Laser epilation emits radiation (F)	85 (21.3)	240 (60.0)	75 (18.8)
3. Laser epilation should not be used during pregnancy (T)	320 (80.0)	20 (5.0)	60 (15.0)
4. Laser epilation should not be used during lactation (T)	270 (67.5)	35 (8.8)	95 (23.8)
5. Laser epilation should not be used for children under 12 years of age (T)	190 (47.5)	85 (21.3)	125 (31.3)
6. Laser epilation may cause skin cancer (F)	50 (12.5)	275 (68.8)	75 (18.8)
7. Laser epilation applied to the genital area may cause infertility (F)	70 (17.5)	245 (61.3)	85 (21.3)
8. Laser epilation applied to the armpit may damage the lymph nodes and cause lymph cancer (F)	75 (18.8)	235 (58.8)	90 (22.5)
9. Laser epilation may cause breast cancer (F)	65 (16.3)	240 (60.0)	95 (23.8)
10. Safety glasses should be used during laser epilation (T)	320 (80.0)	20 (5.0)	60 (15.0)
11. After laser epilation, the application area should be protected from the sun (T)	302 (75.5)	23 (5.8)	75 (18.8)

T: true; F: false; LHR – laser hair removal.
